# The Association Between Asthma and Pancreatic Cancer: A Systematic Review

**DOI:** 10.3390/cancers18213457

**Published:** 2026-10-26

**Authors:** Basel Darawsha, Harish Lavu, Charles J. Yeo, Nader Hanna, Avinoam Nevler

**Affiliations:** Department of Surgery and the Jefferson Pancreas, Biliary and Related Cancer Center, Thomas Jefferson University, Philadelphia, PA 19107, USA

**Keywords:** pancreatic cancer, asthma, atopy, allergy

## Abstract

Background: Pancreatic cancer is an aggressive malignancy, largely due to its vague clinical presentation and delayed diagnosis. Knowledge of the risk factors is essential to combat this disease. Increasing interest has been directed toward immune-related disorders and their effect on pancreatic cancer development. Studies have investigated the relationship between asthma and pancreatic cancer; however, findings are inconsistent, with some studies reporting a protective association, while others failed to detect any differences. In light of these inconsistent findings, we conducted a systematic review of the available literature to clarify the link between asthma and pancreatic cancer. Methods: We identified studies published from 1978 through December 2025 that investigated the association between asthma and pancreatic cancer using PubMed, Scopus, and Embase databases, searched on 2 February 2026. Eligible studies were English-language comparative studies evaluating the association between asthma and pancreatic cancer, including case–control and cohort studies. Eligible outcomes were pancreatic cancer incidence or pancreatic cancer mortality. Studies that did not assess both asthma and pancreatic cancer were excluded. Studies were classified by design, assessed for bias and quality using the Newcastle–Ottawa Scale, and presented as a narrative review. This review was reported in accordance with the Preferred Reporting Items for Systematic Reviews and Meta-Analyses (PRISMA) reporting guideline. Results: A total of 27 studies were included, comprising 14 case–control studies and 13 cohort studies. Overall, the available literature demonstrates discordant findings regarding the association between asthma and pancreatic cancer. While some studies suggest a potential inverse relationship, others report a null or even positive association. Conclusions: The current evidence on the association between asthma and pancreatic cancer remains inconsistent. The discrepancies across studies likely reflect variations in study design, differences in asthma ascertainment, and inconsistent adjustment for established confounders. Future studies with objective asthma diagnosis and adequate confounder control are warranted to clarify this association.

## Simple Summary

Pancreatic cancer remains one of the most aggressive cancers worldwide. There has been growing interest in IgE immune-related disorders, such as asthma, as potential modifiers of pancreatic cancer risk; however, findings have been inconsistent. While some studies, especially those with a case–control design, suggest a possible protective effect of asthma on pancreatic cancer risk, others report no association or even an increased risk of developing or dying from pancreatic cancer. In this systematic review, we identified 27 studies, comprising 14 case–control studies and 13 cohort studies, and narratively summarized their findings and methodology. The inconsistent, and in some cases contradictory, results may be attributed to differences in study design (case–control vs. cohort), how asthma was identified, and adjustment for confounders. Future clinical and laboratory studies are warranted to clarify the true relationship between asthma and pancreatic cancer.

## 1. Introduction

Pancreatic cancer (PC) is a growing global health burden and poses a significant challenge for clinicians, surgeons, oncologists, and health systems worldwide. In 2022, it accounted for nearly 467,000 deaths globally, making it the sixth leading cause of cancer-related death [1]. Although relatively uncommon compared to other types of malignancies, its mortality rate is disproportionately high, with an overall 5-year survival of only 13% [2]. Moreover, epidemiological projection studies estimate that the prevalence of pancreatic cancer will increase by approximately 12.3% by 2040 compared with 2019, further highlighting its public health impact [3]. A thorough understanding of the pathological pathways, including the risk factors that drive PC development, is essential to combating this disease. PC is a multifactorial disease with a range of well-established risk factors. These include environmental exposures such as tobacco use and obesity [4,5], as well as prior medical conditions that significantly contribute to pancreatic carcinogenesis, most notably chronic pancreatitis [6] and diabetes mellitus [7]. In addition, several inherited predisposition genes have been linked to the development of pancreatic cancer, including pathogenic variants in BRCA, ATM, and CDKN2A [8]. Furthermore, multiple hereditary cancer syndromes such as Lynch syndrome and Peutz–Jeghers syndrome have been shown to increase the risk of developing pancreatic cancer [9,10]. Beyond these established risk factors, increasing interest has been directed toward immune-mediated conditions, including asthma, atopic diseases, and allergic disorders (such as food, animal, and other allergies), as potential modifiers of pancreatic cancer risk. Asthma is a chronic immune-mediated obstructive airway disease characterized by bronchial hyper-responsiveness and bronchospasm in response to various allergens. Clinically, it presents with recurrent wheezing, cough, and dyspnea, typically triggered by allergen exposure and environmental factors such as cold air and other stimuli [11]. Similar to other atopic conditions, including atopic dermatitis and allergic rhinitis, the pathogenesis of asthma is driven by type 2 helper T-cell (Th2) immune responses and additional key immunologic components, including immunoglobulin E (IgE), interleukin-4 (IL-4), and the IL-4 receptor α (IL-4Rα) [12]. Previous epidemiologic studies have suggested a possible association between asthma and pancreatic cancer; however, the reported findings have been inconsistent. While some studies have observed an inverse association with pancreatic cancer risk, others have reported no significant difference or even a potential increased risk. In light of these conflicting results, we conducted a PRISMA-compliant systematic review of the available literature to clarify the association between asthma and pancreatic cancer incidence and mortality. Reported findings were systematically assessed for bias and quality using the Newcastle–Ottawa Scale and presented as a narrative review.

## 2. Methods

### 2.1. Selection Process

This review was performed in accordance with the PRISMA (Preferred Reporting Items for Systematic Reviews and Meta-Analyses) guidelines [13]. The review protocol was not registered. Studies published from 1978 to December 2025 were identified through PubMed, Scopus, and Embase. Searches were conducted using the terms “Asthma”, “Atopic diseases”, “Allergy”, “Atopy”, “Cancer”, “Pancreatic neoplasm”, “Malignancy”, and “Pancreatic adenocarcinoma” in multiple combinations on 2 February 2026 (Supplementary Table S1). A single reviewer (B.D.) screened titles and abstracts according to predefined eligibility criteria. The full texts of eligible articles were independently assessed by two authors (B.D. and A.N.). Covidence systematic review software (Veritas Health Innovation, Melbourne, Australia; available at www.covidence.org) was used to manage the screening and selection process.

### 2.2. Eligibility Criteria

Inclusion criteria were English-language publications that employed quantitative comparative study designs, including case–control and cohort studies. Previously published meta-analyses and pooled analyses were identified and discussed separately, but were not counted among the included studies, to avoid double inclusion of the same datasets. Asthma had to be specifically addressed within the definition of atopic disorders, either examined independently or as part of a broader atopy diagnosis. Eligible outcomes were pancreatic cancer incidence or pancreatic cancer mortality. Studies that did not evaluate both pancreatic cancer and asthma were excluded from the review.

### 2.3. Data Extraction

Both authors (B.D. and A.N.) independently assessed the full texts of all eligible studies. Data were extracted into a shared file, including study design, population, number of cases and controls, effect estimates, and adjustment for confounders. Discrepancies between the two authors were resolved by discussion, and the reported data reflect their consensus.

### 2.4. Risk of Bias Assessment

Study quality was assessed using the Newcastle–Ottawa Scale (NOS) [14]. Two authors (B.D. and A.N.) independently performed the assessment, and disagreements were resolved by discussion. Studies scoring 7–9 stars were considered of high quality, 4–6 stars of moderate quality, and 0–3 stars of low quality. The scoring criteria are detailed in Supplementary Material S2.

### 2.5. Data Synthesis

Outcomes were classified as pancreatic cancer incidence or pancreatic cancer mortality and are presented in separate tables. Additionally, studies were categorized according to how asthma was evaluated. Studies that evaluated asthma specifically were classified as Category A, and studies that evaluated asthma as part of a broader atopic phenotype were classified as Category B. The reported associations were categorized as inverse, null, or positive, based on whether the confidence interval (CI) of the adjusted estimate lay entirely below 1.0, included 1.0, or entirely above 1.0, respectively.

A meta-analysis was not performed due to marked heterogeneity in methodology across the included studies, particularly in asthma ascertainment, study design, outcome definition, effect measures, and adjustment for confounders. Therefore, the data were synthesized narratively.

## 3. Results

### 3.1. Study Selection

**Figure 1:**
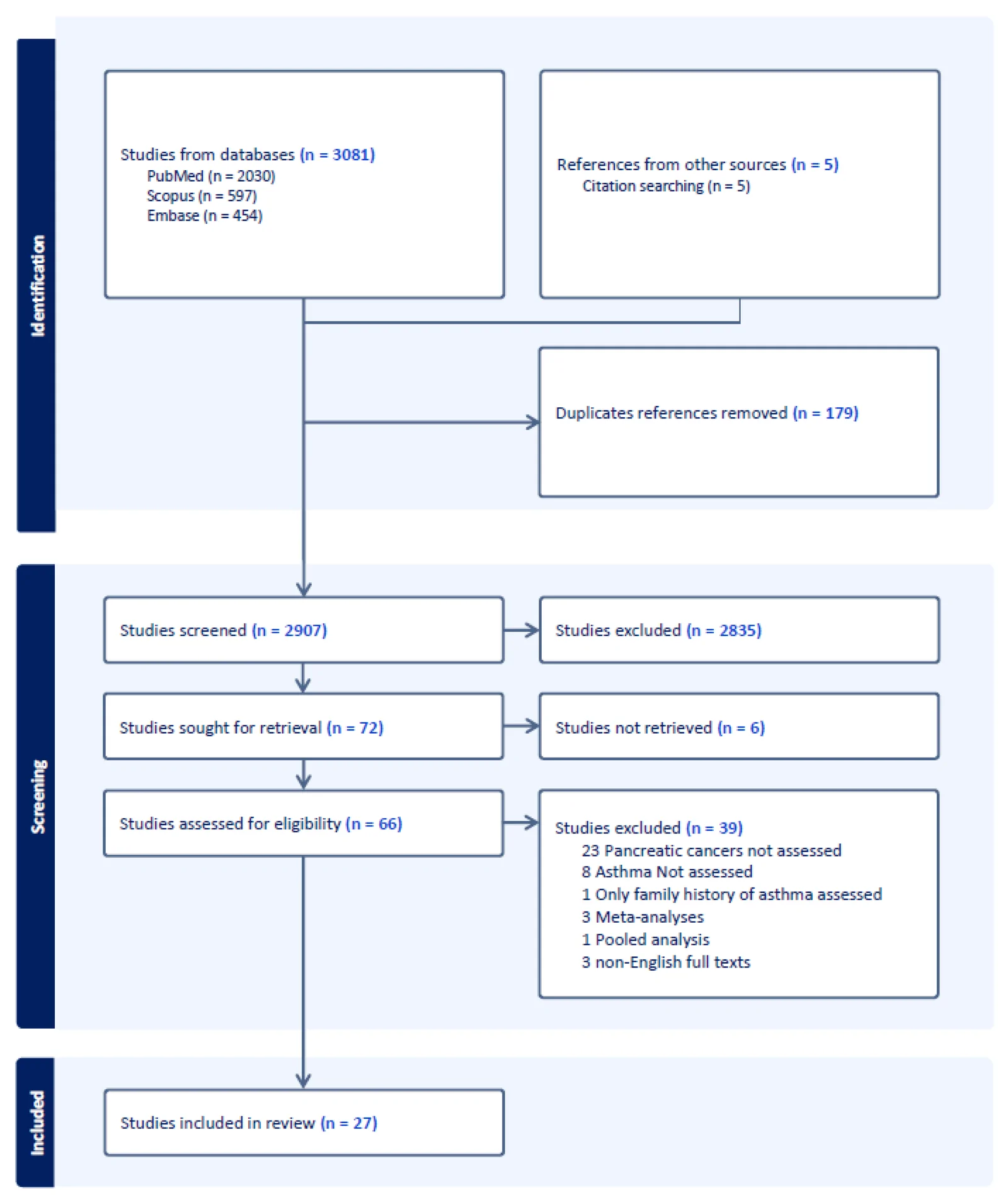
PRISMA 2020 flow diagram.

A total of 3086 studies were identified. After removing 179 duplicates, 2907 studies were screened, and 66 full-text articles were assessed for eligibility. Of these, three meta-analyses and one pooled analysis were discussed separately. A total of 27 primary studies were included: 14 case–control studies and 13 cohort studies. The study selection process is summarized in the PRISMA flow diagram (Figure 1). Additional studies assessing the underlying mechanisms were searched and incorporated into the Introduction and Discussion to clarify the immunologic and pathogenic pathways that may be shared between asthma and pancreatic cancer.

### 3.2. Risk of Bias

According to the Newcastle–Ottawa Scale, 8 studies were of high quality (7–9 stars), 16 of moderate quality (4–6 stars), and 3 of low quality (0–3 stars) (Table 1 and Table 2).

### 3.3. Case–Control Studies

Fourteen case–control studies investigated the association between asthma and pancreatic cancer incidence. Of these, 11 evaluated asthma specifically (Category A), and 3 evaluated asthma as part of a broader atopic phenotype (Category B) (Table 3).

A recent nested case–control study by He et al. (2025) examined the relationship between atopic diseases, type 2 immune effectors, and pancreatic ductal adenocarcinoma (PDAC) using data from the UK Biobank. In this analysis, 544 patients with PDAC were matched 1:4 with 2176 controls based on age, sex, healthcare center, and genetic profile. Asthma was present in 0.9% of individuals in the PDAC group, compared with 2.8% in the control group. This difference corresponded to a 68% reduction in the risk of developing PDAC (odds ratio [OR] 0.32, 95% CI 0.13–0.79). The authors additionally focused on an independent Spanish group comprising 688 participants who were diagnosed with pancreatic cancer and 558 controls. In this group, asthma was also associated with a significantly reduced risk of pancreatic cancer (OR 0.64, 95% CI 0.43–0.92) [15].

A case–control study by Gómez-Rubio et al. (2017; Annals of Oncology), conducted within the PanGenEU consortium, investigated the association between multimorbidity patterns (MPs) and the risk of developing PDAC. In this study, 1705 patients diagnosed with PDAC were compared with 1084 controls. The authors identified three multimorbidity patterns, one of which was an atopic pattern comprising asthma, nasal allergies, and skin allergies. Participants were categorized by the number of atopic conditions diagnosed (0–3), with a score of 3 indicating the presence of all three conditions. A strong correlation was reported between the number of atopic conditions and the decreased risk of PDAC (OR 0.45, 95% CI 0.36–0.55) [16]. An earlier study by Gómez-Rubio et al. (2017; Gut), similarly, based on the PanGenEU Database, investigated the relationship between PDAC and individuals with atopic conditions. A thorough investigation of the clinical aspects of asthma diagnosis included disease severity, asthma medications, duration, and age of onset. 1297 patients with PDAC and 1024 matched controls were identified. Asthma was reported in 88 PDAC cases (6.8%) and 110 controls (10.7%), yielding an OR of 0.64 (95% CI 0.47–0.88). Notably, individuals with long-standing asthma (>17 years) showed a stronger inverse association, with an OR of 0.39. Asthma severity was assessed using definitions from the European Community Health Survey and related questionnaires, with severity scores categorized from 0 to 4 based on clinical manifestations. Participants with an asthma severity score ≥2 had a 51% lower risk of PDAC than those with a severity score of 0 [17].

Other earlier epidemiological case–control studies similarly reported an inverse association between asthma and the development of pancreatic cancer; however, an essential limitation of these studies was that asthma diagnosis relied predominantly on self-reported questionnaires and interviews, without a detailed characterization of disease severity or information regarding medical treatment [18,19,20,21].

Still, the debate regarding the impact of atopic disorders on pancreatic cancer risk persists, as other studies have failed to identify any association [22,23,24,25].

In a case–control study, D’Arcy et al. (2019) investigated the association between allergic diseases (including allergic rhinitis, asthma, and eczema) and overall cancer risk. Asthma was defined using International Classification of Diseases, Ninth Revision (ICD-9) diagnostic codes. The study included 1,744,575 cancer cases and 100,000 controls. Patients with chronic obstructive pulmonary disease (COPD) were excluded from the asthma analysis to control for major confounders such as smoking and to reduce diagnostic misclassification. In the asthma subgroup analysis, the study population consisted of 43,232 pancreatic cancer cases. The primary analysis did not demonstrate an association between asthma and pancreatic cancer (adjusted OR [aOR] 0.94, 95% CI 0.88–1.01). However, in a subgroup analysis requiring at least one claim for asthma medication to classify participants within the asthma group, a mild inverse association between asthma and pancreatic cancer was observed (aOR 0.80, 95% CI 0.69–0.92) [26]. Cotterchio et al. (2014) [27] compared 345 pancreatic cancer cases from the Ontario Cancer Registry with 1285 population-based controls. The asthma group was further stratified into allergic and non-allergic subtypes; neither subtype showed a protective association with PDAC risk, consistent with the author’s prior study [28] (Table 3).

### 3.4. Cohort Studies

Multiple cohort-designed studies have aimed to study the link between asthma and pancreatic cancer. Given their design, these studies provide clearer temporal information and are less prone to recall bias compared with case–control studies. Of the 13 cohort studies included, 10 assessed pancreatic cancer incidence and 3 assessed pancreatic cancer mortality (Table 4 and Table 5).

#### 3.4.1. Asthma and Pancreatic Cancer Incidence

Huang et al. (2018) evaluated atopic allergic conditions, including asthma, in relation to pancreatic cancer risk in a Multiethnic Cohort study [29]. This study included 187,226 participants from five major ethnic groups: White, African American, Latino, Japanese American and Native Hawaiian cohort which followed for a mean duration of 16.2 years. Participants were classified based on self-reported questionnaires, in which individuals were asked whether they had ever been diagnosed with asthma, hay fever, skin allergy, food allergy, or any other allergic condition. Those reporting at least one condition were categorized as having atopic allergic conditions (AACs). Overall, no statistically significant association was observed between a history of AACs and pancreatic cancer risk (relative risk [RR] 1.00, 95% CI 0.88–1.12). Although not statistically significant, a borderline inverse association was noted during the first five years of follow-up (RR 0.76, 95% CI 0.56–1.04), suggesting a potential early protective trend. In subgroup analyses, AACs were associated with a significantly reduced risk of pancreatic cancer among participants aged 70 years or older (RR 0.74, 95% CI 0.56–0.98). A notable limitation in the context of this review is that the number of asthmatic patients in the AAC cohort was not reported. An earlier study from 1988 by McWhorter et al. investigated the relationship between allergies (hay fever, asthma, urticaria, any allergy) and overall risk of cancer and concluded a null association between allergic conditions and pancreatic cancer (risk odds ratio [ROR] 1.69, 95% CI 0.49–5.83). Patients with a diagnosis of asthma were present in 5.7% of the cohort and were not associated with the overall cancer risk (ROR 1.02, 95% CI 0.63–1.65) [30].

In contrast to the studies previously discussed [29,30], which defined exposure based on atopy as a broader phenotype, Guo et al. (2023) evaluated cancer incidence specifically among individuals with asthma using electronic health record data. In this study, 90,021 adults with a documented asthma diagnosis were compared with 270,063 adults without asthma. Regression analyses demonstrated no significant association between asthma or inhaled corticosteroid use and pancreatic cancer risk [31]. A large ICD-10 code-based cohort study by Choi et al. (2023) using data from the Korean National Health Insurance Service assessed 9,892,633 individuals. Overall, no significant association was observed between asthma and pancreatic cancer risk (hazard ratio [HR] 0.98, 95% CI 0.93–1.04). However, sex-stratified analyses showed a modest protective association among allergic males (HR 0.96, 95% CI 0.93–0.99) [32].

A limitation of several previously discussed studies is that definitions of asthma and atopic disease were predominantly based on self-reported questionnaires and ICD diagnostic codes, without objective or quantitative diagnostic assessments. It is also interesting to examine a study by Eriksson et al. (2005) [33], which employed skin-prick testing with multiple inhalant allergens in 13,811 participants examined between 1976 and 1999. Patients with a prior diagnosis of asthma, defined as respiratory symptoms following allergen exposure or evidence of reversible airway obstruction on spirometry, were recruited to the cohort. Additional allergic conditions, including rhinitis, urticaria, and eczema, were also included in the cohort. Among the study population, 2482 individuals were classified as having atopic asthma, while 1955 were diagnosed with non-atopic asthma. Participants were classified into three groups: non-atopic, intermediate, and atopic, with the atopic group defined by having a positive skin prick test to one or more allergens. However, the assessed cohort, even after the follow-up period, was fairly young, and the incidence of pancreatic cancer was extremely low, preventing the detection of any significant association between atopy or allergic symptoms and pancreatic cancer risk. A Finnish study by Vesterinen et al. (1993) evaluated cancer risk among nearly 78,000 patients with asthma and reported no statistically significant relationship between bronchial asthma and pancreatic cancer [34]. Few studies have reported a positive association between asthma and pancreatic cancer risk. The most recent of these is a study by Woo et al. (2021) [35], which analyzed 75,307 propensity score-matched participants using the National Health Insurance Service-based data from South Korea and concluded that a new diagnosis of asthma was associated with a significantly increased risk for pancreatic cancer (HR 1.93, 95% CI 1.16–3.2). However, the study was limited by a younger average age and short follow-up duration, which may limit interpretability. A Swedish study by Liu et al. (2015) [36] investigated the association between distinct asthma presentations and multiple cancer types, including pancreatic cancer. The asthma cohort was stratified into predominantly allergic asthma, non-allergic asthma, unspecified asthma, and status asthmaticus. Statistical analysis yielded a standardized incidence ratio (SIR) of 1.35 (95% CI 1.19–1.52) for pancreatic cancer among the overall asthma population. The highest risk was observed in the unspecified asthma subgroup (SIR 1.37, 95% CI 1.17–1.60), whereas no significant association was found for the remaining phenotypes. However, this study did not exclude patients with COPD or other obstructive lung diseases that may overlap with asthma, which may represent an important confounder.

Earlier studies reporting a positive association were conducted in a highly specific subpopulation, which significantly limits their generalizability. For example, a study by Ji et al. (2009) [37] reported an SIR of 1.56 (95% CI 1.36–1.78) for pancreatic cancer risk among participants who were hospitalized for asthma between 1965 and 2004 in Sweden. Another study within the Alpha-Tocopherol Beta-Carotene Cancer Prevention (ATBC) Study reported a positive association between pancreatic cancer risk and a self-reported history of asthma among male smokers (HR 2.16, 95% CI 1.17–3.98) [38] (Table 4).

**Table 4:** Cohort studies examining the association between asthma and pancreatic cancer incidence.

Author (Year Published) [Ref]	Period	Country	Follow-Up (Years) ^1^	Asthma Ascertainment	Cohort Size	Adjusted for Smoking	Association	Effect Estimate (95% CI)	Category ^2^
Guo (2023) [31]	2012–2020	USA	1.1	ICD-9/10	360,084	Yes	Null	HR 1.03 (0.73–1.45) ^3^	A
Choi (2023) [32]	2009–2017	Korea	7.3 [7.10–7.58]	ICD-10	9,892,633	Yes	Null	HR 0.98 (0.93–1.04)	A
Huang (2018) [29]	1993–2012	California, Hawaii, USA	16.2 ± 4.7	Questionnaires	187,226	Yes	Null	RR 1.00 (0.88–1.12)	B
Eriksson (2005) [33]	1976–1999	Sweden	9.5 [1.5–20] ^4^	Skin prick test	13,811	No	Null	SIR 0.72 (0.09–2.60)	B
Vesterinen (1993) [34]	1970–1987	Finland	17	Pulmonary function and/or Skin test	35,126 Males	No	Null	SIR 0.88 (0.67–1.13)	A
					42,826 Females	No	Null	SIR 1.01 (0.8–1.24)	A
McWhorter (1988) [30]	1971–1984	USA	8.97	Questionnaires	6108	Yes	Null	ROR 1.69 (0.49–5.83)	B
Woo (2021) [35]	2002–2015	Korea (NHIS-NSC)	7.60 ± 3.74	ICD-10	75,307 ^5^	Yes	Positive	HR 1.93 (1.16–3.2)	A
Liu (2015) [36]	1987–2010	Sweden	4 [1,2,3,4,5,6,7]	ICD-9/10	439,455	No	Positive	SIR 1.35 (1.19–1.52)	A
Ji (2009) [37]	1965–2004	Sweden	Up to 40	ICD-7/8/9/10	140,425	No	Positive	SIR 1.56 (1.36–1.78)	A
Stolzenberg-Solomon (2002) [38]	1985–1997	Finland	10.2 (median; maximum 13)	Questionnaires	29,048	Yes	Positive	HR 2.16 (1.17–3.98)	A

Abbreviations: CI, confidence interval; HR, hazard ratio; ICD, International Classification of Diseases; NHIS-NSC, National Health Insurance Service-National Sample Cohort; ROR, risk odds ratio; RR, relative risk; SIR, standardized incidence ratio; IQR, interquartile range; SD, standard deviation. ^1^ Years are reported as Mean± SD, Median [IQR], or total follow-up as presented in the original study. ^2^ Category A: studies that evaluated asthma specifically; Category B: studies that evaluated asthma as part of a broader atopic phenotype. ^3^ 99% confidence interval. ^4^ [10–90th percentile]. ^5^ Propensity score-matched cohort drawn from 475,197 participants.

#### 3.4.2. Asthma and Pancreatic Cancer Mortality

Three cohort studies evaluated the association between asthma and pancreatic cancer mortality, all of which assessed asthma specifically (Category A); Turner et al. additionally reported an estimate for participants with both asthma and hay fever (Category B). While some studies report no significant association [39], others suggest an increased risk of pancreatic cancer mortality among individuals with asthma [40]. A notable study by Turner et al. (2005) [41] stratified participants according to self-reported diagnoses of asthma, hay fever, or both, and compared them with individuals without these conditions. Relative risks for site-specific cancer mortality were calculated. As for pancreatic cancer, no significant association was observed among individuals with asthma alone (RR 1.02, 95% CI 0.85–1.23) or those with both asthma and hay fever (RR 1.14, 95% CI 0.94–1.38) (Table 5).

**Table 5:** Cohort studies examining the association between asthma and pancreatic cancer mortality.

Author (Year Published) [Ref]	Period	Country	Follow-Up (Years) ^1^	Asthma Ascertainment	Cohort Size	Adjusted for Smoking	Association	Effect Estimate (95% CI)	Category ^2^
Turner (2005) [41]	1982–2000	USA	18	Questionnaires	1,102,247	Yes	Null	RR 1.02 (0.85–1.23)	A
								RR 1.14 (0.94–1.38) ^3^	B
Mills (1988) [39]	1976–1982	USA	6	Questionnaires	34,198	No	Null	RR 0.87 (0.21–3.63)	A
Robinette (1978) [40]	1944–1974	USA	29	Army hospital diagnosis	19,100	No	Positive	3.4-fold *p* value ≤ 0.05 ^4^	A

Abbreviations: CI, confidence interval; RR, risk ratio. ^1^ Total follow-up period. ^2^ Category A: studies that evaluated asthma specifically; Category B: studies that evaluated asthma as part of a broader atopic phenotype. ^3^ Participants reporting both asthma and hay fever, compared with those reporting neither asthma nor hay fever. ^4^ Ratio of observed pancreatic cancer deaths in the asthma group vs. the control group (17 vs. 5 deaths); *p* value ≤ 0.05; no confidence interval reported.

## 4. Previous Meta-Analyses

The inconsistent and, in some cases, conflicting conclusions described above stem from multiple factors, including heterogeneity in study design, limited sample size, variability in exposure and severity assessment, and different approaches to handling confounders such as smoking and past medical history. To address these limitations, meta-analyses are important because they increase statistical power by pooling data from broader, more diverse study populations, thereby improving generalizability.

A recent meta-analysis by Guo et al. (2025) [42] evaluated 53 studies published in the past 20 years that examined the association between allergic conditions and cancer risk. The authors identified 26 terms across all included articles that refer to allergy and grouped them into 7 allergy subtypes, including asthma. Overall, an inverse association between allergic conditions and pancreatic cancer was observed (OR 0.85, 95% CI 0.79–0.92). However, subgroup analyses did not show a statistically significant association between pancreatic cancer risk and asthma (OR 0.89, 95% CI 0.65–1.23).

Similarly, an earlier meta-analysis by Gandini et al. (2005) [43] evaluated 14 population-based studies (4 cohort and 10 case–control) published between 1981 and 2003, totaling 3040 pancreatic cancer cases. While allergic conditions overall were associated with a reduced risk of pancreatic cancer, analyses restricted to asthma showed no association (RR 1.01, 95% CI 0.77–1.31). Notably, Gómez-Rubio et al. (2017; Gut) [17] conducted a meta-analysis of 10 case–control studies published between 1986 and 2014 and reported a statistically significant inverse association between asthma and pancreatic ductal adenocarcinoma (OR 0.73, 95% CI 0.59–0.89). This analysis showed lower heterogeneity among studies (I^2^ = 13.5%) than in previously discussed meta-analyses, likely due to the restriction to a single study design.

Olson et al. (2013) conducted a pooled analysis of 10 case–control studies, including 3567 pancreatic cancer cases and 9145 controls. Among the six studies that specifically evaluated asthma in addition to other allergic exposures, no significant association with pancreatic cancer was observed (OR 0.91, 95% CI 0.64–1.30) [44].

## 5. Discussion

The inter-relationship between asthma and other atopic disorders and pancreatic cancer has been the focus of numerous studies over the past decades. The changes in the terminology of lung disease and the changing trends in smoking prevalence further complicate our ability to assess studies for earlier time periods. While the relationship between asthma and pancreatic cancer remains largely uncertain, this review aims to highlight the main studies that investigated this possible association and notes the disparate findings.

In this systematic review of 27 studies, seven studies reported an inverse association between asthma and pancreatic cancer risk [15,16,17,18,19,20,21], fifteen studies report findings of no significant association [22,23,24,25,26,27,28,29,30,31,32,33,34,39,41] and five reported positive associations [35,36,37,38,40]. Although multiple immunologic hypotheses have been proposed to explain a possible link between asthma and pancreatic cancer, none has provided a definitive explanation, as biological evidence exists supporting either hypothesis.

Understandably, some of the discordant findings may be due to differences in study design. Case–control studies are susceptible to recall bias and survivor bias, as many studies excluded a large proportion of cases due to early mortality, non-response to questionnaires, or other challenges in participant recruitment. Cohort studies, on the other hand, frequently rely on administrative and hospital records, in which asthma may be misclassified, and are susceptible to surveillance bias, as individuals with asthma receive more medical attention than those without asthma. Additionally, discrepancies in asthma ascertainment significantly contribute to the mixed results, as some studies relied on self-reported questionnaires and others used medical interviews and objective diagnostic tools.

Differences in confounder control across studies may further explain the discrepant findings. For example, not all included studies adjusted for smoking, a well-established risk factor for pancreatic cancer. Inadequate adjustment for other confounders, such as BMI, baseline comorbidities, and medication use, may also have affected the reported results. Reverse causation is an additional concern, as early undiagnosed pancreatic cancer may present with nonspecific and vague symptoms, prompting patients to seek medical attention before formal diagnosis. For example, in a Swedish group of patients hospitalized for asthma, Ji et al. (2009) [37] reported an SIR for pancreatic cancer of 2.90 (95% CI 2.10–3.89) in the first year after hospitalization, which decreased to 1.50 (95% CI 1.13–1.95) among patients whose last asthma hospitalization occurred 5–9 years earlier, a pattern consistent with reverse causation. In an attempt to reduce this bias, D’Arcy et al. (2019) [26] excluded asthma claims recorded in the year before cancer diagnosis.

Although differences in methodology may account for part of the inconsistent findings, exploration of the underlying biological mechanisms is essential for a more comprehensive understanding of the link between asthma and pancreatic cancer.

Several biological pathways and immunologic mediators have been proposed to be shared in the pathophysiology of asthma and pancreatic cancer. Among these factors are IgE antibodies, which play a central role in the pathogenesis of asthma and other atopic diseases [45,46]. Elevated serum IgE levels have also been associated with a reduced risk of developing certain malignancies [47]. Experimental studies have demonstrated antitumor activity of chimeric IgE antibodies in ovarian and colorectal cancer models [48,49]. Furthermore, Fu et al. demonstrated in vitro antitumor activity of IgE antibodies against pancreatic cancer cells [50]. Overall, reports from the emerging field of allergo-oncology suggest that higher IgE levels may play a protective role in pancreatic cancer development [51]. Conversely, He et al. [15] measured IgE levels in 688 patients with pancreatic cancer and 558 controls from the PanGenEU study and found that the inverse association between asthma and PDAC was present only among individuals with low IgE sensitization, whereas no significant association was observed among those with high IgE sensitization, defined as total IgE > 100 kU/L. Other studies have suggested that allergen binding to IgE may compromise the capacity of these antibodies to eliminate neoplastic cells by interfering with their binding to tumor-specific antigens [52]. Nonetheless, additional studies that have contributed to the ambiguity surrounding the role of IgE in cancer pathogenesis have reported that allergic conditions do not necessarily confer a protective effect and are not associated with an increased risk of malignancy [53].

Beyond allergo-oncologic mechanisms, additional biological domains have been proposed to contribute to the ongoing debate surrounding the association between asthma and pancreatic cancer, including alterations in the microbiome. Several studies describe a complex interplay between asthma, pancreatic cancer, and the microbiome. These studies have reported that Porphyromonas gingivalis, a Gram-negative obligate anaerobe and a predominant component of the human oral microbiota [54], may confer a protective effect against allergic conditions, including asthma [55]. In contrast, P. gingivalis has been associated with an increased incidence of pancreatic cancer, potentially through acceleration of progression from early pancreatic intraepithelial neoplasia to adenocarcinoma [56,57].

Immune surveillance and chronic inflammation are two contrasting immunologic hypotheses proposed to explain the shared pathophysiology of asthma and pancreatic cancer. The immune surveillance hypothesis suggests that atopic diseases, including asthma, may act as a protective factor against pancreatic cancer. According to this hypothesis, the immune system is continuously stimulated by environmental allergens, resulting in a state of hypersensitivity that enhances immune surveillance and enables the recognition and elimination of neoplastic cells [43,52,58,59,60,61]. Conversely, the chronic inflammation hypothesis suggests that allergic diseases involve sustained immune activation, creating a persistent inflammatory microenvironment that may promote oxidative tissue damage, compromise DNA repair, and thereby facilitate carcinogenesis [15,62]. Nevertheless, this mechanism has been more consistently linked to the increased risk of lung cancer among patients with asthma than to pancreatic cancer [63,64].

A limitation of this systematic review is that the initial screening was conducted by a single reviewer. Although final eligibility was subsequently assessed by a second reviewer, single-reviewer screening may have introduced selection bias. Additionally, all studies included in this review are observational and therefore establish association rather than biological causation.

Some eligible studies were identified through manual review of the reference list of included articles, suggesting that the search strategy may not have captured all relevant studies. Therefore, it remains possible that other eligible studies were not retrieved. In addition, the inclusion of only English-published articles may have led to the omission of relevant non-English publications. Finally, the study protocol was not registered; however, the outcomes were defined before the search was conducted.

## 6. Conclusions

The available evidence is inconsistent and does not establish a clear association between asthma and pancreatic cancer. Further research is warranted to investigate this relationship and the potential underlying immune-related pathways.

## Figures and Tables

**Table 1:** Risk of bias assessment of the included case–control studies using the Newcastle–Ottawa Scale.

Study [Ref]	Selection	Comparability	Exposure	Total	Quality
He et al., 2025 [15]	★★★☆	★★	★☆☆	6/9	Moderate
Gómez-Rubio et al., 2017 (Ann Oncol) [16]	★★★☆	★★	★☆☆	6/9	Moderate
Gómez-Rubio et al., 2017 (Gut) [17]	★★★☆	★★	★★☆	7/9	High
Maisonneuve et al., 2010 [18]	★★☆☆	★★	★☆☆	5/9	Moderate
Holly et al., 2003 [19]	★★☆☆	☆☆	★☆☆	3/9	Low
Bueno de Mesquita et al., 1992 [20]	★★★☆	★☆	★☆☆	5/9	Moderate
Mack et al., 1986 [21]	★★★☆	☆☆	★☆☆	4/9	Moderate
Silverman et al., 1999 [22]	★★☆☆	★★	★★★	7/9	High
Dai et al., 1995 [23]	★★☆☆	★☆	★☆☆	4/9	Moderate
Farrow et al., 1990 [24]	★★☆☆	☆☆	★☆☆	3/9	Low
Jain et al., 1991 [25]	★★★☆	★☆	★☆☆	5/9	Moderate
D’Arcy et al., 2019 [26]	★★★☆	☆☆	★★★	6/9	Moderate
Cotterchio et al., 2014 [27]	★★☆☆	★★	★☆☆	5/9	Moderate
Eppel et al., 2007 [28]	★★★☆	☆☆	★☆☆	4/9	Moderate

Studies scoring 7–9, 4–6, and 0–3 stars were considered of high, moderate, and low quality.

**Table 2:** Risk of bias assessment of the included cohort studies using the Newcastle–Ottawa Scale.

Study [Ref]	Selection	Comparability	Outcome	Total	Quality
Huang et al., 2018 [29]	★★★☆	★★	★★★	8/9	High
McWhorter et al., 1988 [30]	★★★★	★☆	★★☆	7/9	High
Guo et al., 2023 [31]	★★★★	★★	★☆☆	7/9	High
Choi et al., 2023 [32]	★★★★	★★	★★☆	8/9	High
Eriksson et al., 2005 [33]	★★★☆	☆☆	★★☆	5/9	Moderate
Vesterinen et al., 1993 [34]	★★★☆	☆☆	★★☆	5/9	Moderate
Woo et al., 2021 [35]	★★★★	★★	★☆☆	7/9	High
Liu et al., 2015 [36]	★★★★	☆☆	★★☆	6/9	Moderate
Ji et al., 2009 [37]	★★☆☆	☆☆	★★★	5/9	Moderate
Stolzenberg-Solomon et al., 2002 [38]	★★☆☆	★☆	★★★	6/9	Moderate
Mills et al., 1988 [39]	★☆☆☆	☆☆	★☆☆	2/9	Low
Robinette et al., 1978 [40]	★★☆☆	☆☆	★★★	5/9	Moderate
Turner et al., 2005 [41]	★★☆☆	★★	★★★	7/9	High

Studies scoring 7–9, 4–6, and 0–3 stars were considered of high, moderate, and low quality.

**Table 3:** Case–control studies examining the association between asthma and pancreatic cancer incidence.

Author (Year Published) [Ref]	Period	Country	Asthma Ascertainment	Cases/Controls	Adjusted for Smoking	Association	Effect Estimate (95% CI)	Category ^1^
He (2025) He (2025) [15]	From 2006 2009–2014; 2016–2018	United Kingdom (Biobank) Spain (PanGenEU)	Doctor-diagnosed asthma Interviews and Questionnaire	544/2176 688/558	Yes Yes	Inverse Inverse	OR 0.32 (0.13–0.79) OR 0.64 (0.43–0.92)	A A
Gómez-Rubio (2017), Ann Oncol [16]	2009–2014	England, Germany, Ireland, Italy, Spain, Sweden (PanGenEU)	Interviews and Questionnaire	1705/1084	Yes	Inverse	OR 0.45 (0.36–0.55) ^2^	B
Gómez-Rubio (2017), Gut [17]	2009–2014	England, Germany, Ireland, Spain (PanGenEU)	Interviews and Questionnaire	1297/1024	Yes	Inverse	OR 0.64 (0.47–0.88)	A
Maisonneuve (2010) [18]	1983–1988	Canada, The Netherlands, Poland, Australia	Interviews and Questionnaire	823/1679	Yes	Inverse	OR 0.57 (0.34–0.97)	A
Holly (2003) [19]	1994–2001	San Francisco Bay Area, USA	Interviews and Questionnaire	532/1701	No	Inverse	OR 0.53 (0.37–0.75)	B
Bueno de Mesquita (1992) [20]	1984–1988	The Netherlands	Interviews and Questionnaire	176/487	Yes	Inverse	OR 0.57 (0.36–0.9)	B
Mack (1986) [21]	1976–1981	Los Angeles County, USA	Interviews and Questionnaires	124/124	No	Inverse	RR 0.2 (0.1–0.8) ^3^	A
Jain (1991) [25]	1983–1986	Canada	Interviews and Questionnaires	249/505	Yes	Null	RR 0.52 (0.16–1.70)	A
D’Arcy (2019) [26]	1992–2013	USA	ICD-9	43,232/81,234	No—Patients with COPD were excluded.	Null ^4^	OR 0.94 (0.88–1.01)	A
Cotterchio (2014) [27]	2011–2012	Canada	Questionnaires	345/1285	Yes	Null	OR 0.89 (0.60–1.30)	A
Eppel (2007) [28]	2003–2006	Canada	Questionnaires	276/378	No	Null	OR 0.6 (0.31–1.16)	A
Silverman (1999) [22]	1986–1989	Atlanta, Detroit, New Jersey, USA	Interviews and Questionnaires	484/2099	Yes	Null	OR 1.0 (0.6–1.5)	A
Dai (1995) [23]	1992–1993	China	Interviews and Questionnaires	108/275	Yes	Null	OR 1.0 (0.3–3.2)	A
Farrow (1990) [24]	1982–1986	Washington, USA	Interviews and Questionnaires	148/188	No	Null	OR 1.1 (0.4–3.2)	A

Abbreviations: CI, confidence interval; OR, odds ratio; RR, risk ratio; COPD, chronic obstructive pulmonary disease; ICD, International Classification of Diseases. ^1^ Category A: studies that evaluated asthma specifically; Category B: studies that evaluated asthma as part of a broader atopic phenotype. ^2^ High adherence to the Atopic Multimorbidity Pattern compared with low adherence. ^3^ Among 124 pairs who were directly interviewed, analysis of all 490 pairs, including proxy interviews, yielded a risk ratio of 0.7 (95% CI, 0.4–1.1). ^4^ Sensitivity analysis requiring ≥1 asthma medication claim reported an inverse association between asthma and pancreatic cancer (OR 0.80, 95% CI, 0.69–0.92).

## Data Availability

All data supporting the findings are available in the cited publications and the Supplementary Materials.

## Supplementary Materials

The following supporting information can be downloaded at: https://www.mdpi.com/article/10.3390/cancers18213457/s1, Table S1, Search strategies used for each database; Material S2, Newcastle–Ottawa Quality Assessment Scale, Material S3, PRISMA 2020 Main Checklist.
