# Supplementary material for: The Association Between Asthma and Pancreatic Cancer: A Systematic Review
Source: Cancers (Basel). 2026 Oct 26;18(21):3457. doi: 10.3390/cancers18213457 (PMC13761428; doi:10.3390/cancers18213457)
Supplement: Supplementary file 1 [file cancers-18-03457-s001.zip › Supplementary Material S2.pdf]

Supplementary Material S2. NEWCASTLE - OTTAWA QUALITY ASSESSMENT SCALE [1]

CASE CONTROL STUDIES

Selection

1) Is the case definition adequate?
   a) yes, with independent validation ★
   b) yes, e.g., (record linkage or based on self-reports)
   c) no description

2) Representativeness of the cases
   a) consecutive or obviously representative series of cases ★
   b) potential for selection biases or not stated

3) Selection of Controls
   a) community controls ★
   b) hospital controls
   c) no description

4) Definition of Controls
   a) no history of disease (endpoint) ★
   b) no description of source

Comparability

1) Comparability of cases and controls on the basis of the design or analysis
   a) study controls for **age and smoking** ★
   b) study controls for any additional factor – **Diabetes or BMI** ★

Exposure

1) Ascertainment of exposure
   a) secure record ★
   b) structured interview where blind to case/control status ★
   c) interview not blinded to case/control status
   d) written self-report or medical record only
   e) no description

2) Same method of ascertainment for cases and controls
   a) yes ★
   b) no

3) Non-Response rate
   a) same rate for both groups ★
   b) non respondents described
   c) rate different and no designation

COHORT STUDIES

Selection

1) Representativeness of the exposed cohort
   a) truly representative of the average (**adults with asthma)** in the community ★
   b) somewhat representative of the average (**adults with asthma)** in the community ★
   c) selected group of users e.g., nurses, volunteers
   d) no description of the derivation of the cohort

2) Selection of the non-exposed cohort
   a) drawn from the same community as the exposed cohort ★
   b) drawn from a different source
   c) no description of the derivation of the non-exposed cohort

3) Ascertainment of exposure
   a) secure record (e.g., medical records) ★
   b) structured interview ★
   c) written self-report
   d) no description

4) Demonstration that outcome of interest was not present at start of study
   a) yes ★
   b) no

Comparability

1) Comparability of cohorts on the basis of the design or analysis
   a) study controls for (**age and smoking)** (select the most important factor) ★
   b) study controls for any additional factor (**BMI or Diabetes**) ★

Outcome

1) Assessment of outcome
   a) independent blind assessment ★
   b) record linkage ★
   c) self-report
   d) no description

2) Was follow-up long enough for outcomes to occur
   a) yes ★
   b) no

3) Adequacy of follow up of cohorts
   a) complete follow up - all subjects accounted for ★
   b) subjects lost to follow up unlikely to introduce bias - small number lost - **70% follow up, or description provided of those lost** ★
   c) follow up rate < **30% and no description of those lost**
   d) no statement

1. Wells, G.A.S., B.; O'Connell, D.; Peterson, J.; Welch, V.; Losos, M.; Tugwell, P. The Newcastle-Ottawa Scale (NOS) for Assessing the Quality of Nonrandomised Studies in Meta-Analyses. Available online: https://ohri.ca/en/who-we-are/core-facilities-and-platforms/ottawa-methods-centre/newcastle-ottawa-scale (accessed on
