# Supplementary material for: The Association Between Asthma and Pancreatic Cancer: A Systematic Review
Source: Cancers (Basel). 2026 Oct 26;18(21):3457. doi: 10.3390/cancers18213457 (PMC13761428; doi:10.3390/cancers18213457)
Supplement: Supplementary file 1 [file cancers-18-03457-s001.zip › TableS1.pdf]

Table S1. Search strategies used for each database.

Database	Search string	Filters	Records identified
**PubMed**	("asthma"[Title/Abstract] OR "atopic diseases"[Title/Abstract] OR "allergy"[Title/Abstract] OR "atopy"[Title/Abstract]) AND ("cancer"[Title/Abstract] OR "pancreatic neoplasm"[Title/Abstract] OR "malignancy"[Title/Abstract] OR "pancreatic adenocarcinoma"[Title/Abstract])	English, Adults	2,030
**Embase**	('asthma':ti OR 'atopic diseases':ti OR 'allergy':ti OR 'atopy':ti) AND ('cancer':ti OR 'pancreatic neoplasm':ti OR 'malignancy':ti OR 'pancreatic adenocarcinoma':ti)	Non	454
**Scopus**	TITLE-ABS-KEY ((Asthma OR atopy OR allergy) AND (Cancer OR pancreatic neoplasm OR malignancy OR pancreatic adenocarcinoma))	Non	597
